# Correlation of BRAF Variant V595E, Breed, Histological Grade and Cyclooxygenase-2 Expression in Canine Transitional Cell Carcinomas

**DOI:** 10.3390/vetsci6010031

**Published:** 2019-03-19

**Authors:** Julia M. Grassinger, Sophie Merz, Heike Aupperle-Lellbach, Hanna Erhard, Robert Klopfleisch

**Affiliations:** 1 Laboklin GmbH & Co. KG, 97688 Bad Kissingen, Germany; aupperle@laboklin.com (H.A.-L.); h.erhard@laboklin.com (H.E.); 2 Institute of Veterinary Pathology, Freie Universität Berlin, 14163 Berlin, Germany; Sophie.Merz@fu-berlin.de (S.M.); Robert.Klopfleisch@fu-berlin.de (R.K.)

**Keywords:** dog, terrier, urothelial carcinoma, histological grading, BRAF mutation, COX-2

## Abstract

The presence of BRAF variant V595E, as well as an increased cyclooxygenase-2 (COX-2) expression in canine transitional cell carcinoma (TCC) are well-described in the literature. The aim of the present study was to investigate the correlation between breed (terrier versus non-terrier dogs), histological grade, COX-2 expression, and BRAF mutation in canine TCC. Therefore, transmural TCC biopsies from 65 dogs (15 terriers, 50 non-terriers) were graded histologically into low- and high-grade. Immunohistochemical evaluation of the intensity of COX-2 expression was performed using an immunoreactive score (IRS). Exon 15 of chromosome 16 was examined for the BRAF variant c.1799T>A by TaqMan^®^ SNP assay. TCC was low-grade in 20 cases (one terrier, 19 non-terriers) and high-grade in 45 cases (14 terriers, 31 non-terriers). Contrary to humans, histological grade was not significantly correlated to the intensity of COX-2 expression. BRAF mutation was detected in 11/15 (73%) TCC of terriers and in 18/50 (36%) TCC of non-terriers. Histological grade and BRAF mutation were not correlated significantly (*p* = 0.2912). Terriers had a considerably higher prevalence of high-grade tumors (*p* < 0.0001), as well as of BRAF mutation (*p* ≤ 0.05) compared to non-terriers. In non-terriers, neoplasms with BRAF mutation showed a significantly higher intensity of COX-2 expression than those without BRAF mutation (*p* ≤ 0.05). In conclusion, in contrast to humans, testing for BRAF mutation in canine TCC is a sensitive diagnostic method especially in terriers (73%) and may be recommended as a screening test. However, evidence of BRAF mutation in canine TCC is not a predictor for the histological grade. Moreover, a positive correlation between histological grade and the intensity of COX-2 expression was not found. Further studies are necessary to clarify the clinical and prognostic relevance of the elevated intensity of COX-2 expression of TCC with BRAF mutation detected in non-terriers.

## 1. Introduction

Transitional cell carcinoma (TCC), also referred to as urothelial carcinoma, is the most common malignant tumor of the canine urinary tract. The breed-associated risk includes a 21-fold increased risk in Scottish terriers and a 3.0–6.5-fold increased risk in Eskimo dogs, Shetland sheepdogs, West Highland white terriers, keeshonds, samoyeds, and beagles compared to mixed breed dogs [1]. The mean age of dogs at time of diagnosis is 11 years [2]. Several studies have confirmed the increased risk of bladder cancer in female dogs and in neutered ones compared to intact dogs of the same gender [3]. Interestingly, in dogs with high breed-associated risk, the sex predilection is less pronounced [4]. High-grade TCC is reported in more than 90% of dogs suffering from TCC [2]. Metastasis at the time of diagnosis is described in 37% of dogs [5]. In dogs suffering from TCC that only undergo surgical therapy, a longer mean survival time has rarely been observed, in contrast to dogs with TCC that have been treated with additional therapies [6]. Thus, other treatments are recommended, such as radiotherapy, transurethral diode laser resection, intralesional interleukin-2 injection, classic chemotherapy, or metronomic chemotherapy with continuous oral administration of chlorambucil [6]. Knapp et al. reported that the nonselective cyclooxygenase (COX) inhibitor piroxicam significantly enhanced the activity of vinblastine, a chemotherapeutic agent, in dogs with TCC [7]. However, the complex mechanisms of the effect of COX inhibitors are still poorly understood [8]. Mohammed et al. [9] reported a reduction in tumor volume in 12 of 18 dogs due to induction of apoptosis caused by piroxicam. COX enzymes catalyze the rate-limiting step of prostaglandin biosynthesis and are involved in inhibiting apoptosis, promoting cell proliferation, stimulating angiogenesis, and decreasing immunity [10]. In general, COX-1 is present in several normal tissues, and COX-2 is expressed in multiple neoplasms and inflammatory cells [11]. Regular expression of COX-1 has been described in normal urinary bladder epithelium, but intense COX-2 expression was found in canine TCC [12]. There are different examination methods to evaluate COX-2 expression: Khan et al. estimated the percentage of COX-2 positive neoplastic cells and their staining intensity, but did not use a scoring system [12]. Lee et al. reported increased COX-2 expression in 57.7% of canine TCC based only on the percentage of positive neoplastic cells, but not including the various expression intensities within one TCC [13]. Knottenbelt et al. reported positive COX-2 expression in up to 100% of the canine TCC examined considering the different expression intensities and percentages of positive cells within one TCC, but without calculating any scores nor describing whether neoplastic and/or inflammatory cells were taken into account [8]. Mutsaers et al. [14] did not believe the intensity of COX-2 expression was a useful predictive factor for a response to piroxicam. In his study, he included the percentage of neoplastic cells with COX-2 immunoreactivity, as well as the different staining intensities, but did not work with any scoring system. Furthermore, he only evaluated the area of the highest COX-2 staining (“hot spot”) without considering areas with lower COX-2 expression. This may have caused an overestimation of the COX-2 expression in the investigated TCC and could be the reason for the low response to piroxicam that was observed, even though COX-2 expression was high. In vitro, the selective COX-2 inhibitor mavacoxib exerts antitumor effects independent of COX-2 expression levels in canine, as well as in human cell lines [15]. However, in vivo studies addressing this finding are not available so far. The different studies regarding the intensity of COX-2 expression are therefore not comparable and should be interpreted carefully.

In humans, 2% of all cancers are TCC [2]. The mean age at time of diagnosis is 65 years [2], and a gender predisposition is described for men [16]. The majority of bladder cancers in humans are classified as superficial low-grade TCC [3]. However, in invasive high-grade TCC, metastasis is reported in 5–20% of cases at the time of diagnosis [16]. The COX-2 expression in humans is a marker of invasion, recurrence, and a short survival time [17,18,19]. It is particularly expressed in high-grade forms, possibly playing a role in the differentiation of this tumor and being a valuable target molecule in the treatment of TCC [20]. In the standard treatment of human TCC, transurethral resection is performed first [21]. Post-transurethral resection, intravesical therapy with bacillus Calmette-Guérin, an attenuated mycobacterium with antitumor activity when administered as an intravesical instillation [22], has been shown to reduce the risk of recurrence. Alternative chemotherapy agents, such as mitomycin C, doxorubicin, epirubicin, gemcitabine, and thiotepa, can be used for intravesical instillation [23]. For patients with inoperable, locally-advanced metastatic TCC, systemic chemotherapy is the standard initial treatment [24]. In the second-line setting, immunotherapy with immune check point inhibitors is now the standard of care [24].

Mutations of BRAF genes (B-isoform of rapidly accelerated fibrosarcoma) are a common cause of tumor formation in humans and lead to abnormal proliferation and differentiation of cells [25]. Studies from the U.S. have shown that BRAF variant V595E (cBRAF reference sequence ENSCAFT00000006306), which corresponds to the BRAF (V600E) variant in humans, can be found in tumor cells of 65–85% of dogs with TCC [26,27,28,29]. In humans, as well as in dogs, BRAF mutation is a thymine-to-adenine transversion in exon 15 of chromosome 16, resulting in the amino acid substitution from valine to glutamic acid [30]. This somatic mutation was specifically found in canine TCC and prostate tumors, but rarely in other canine tumors [31]. A breed predisposition showing a BRAF mutation in cases of TCC was found for terrier breeds [6]. In humans, only 1% of TCC is caused by BRAF mutation [32], and this mutation appeared to be infrequent in TCC [31]. However, BRAF mutation plays a crucial role in human cancer, and about 7% of all cancer cases carry this mutation [33]. It is frequently mutated in melanomas (50%) [34], papillary thyroid cancers (45%) [35], colon cancers (10%) [36], and in non-small-cell lung cancers (10%) [37]. A positive correlation of COX-2 expression and the presence of BRAF mutation were reported in human colorectal cancer [38], but have not been investigated in dogs. Therapeutic concepts against human tumors induced by BRAF mutation include MAP kinase inhibitors, which are used in human medicine [39]. Bourn and Cekanova reported a potentiated MAP kinase inhibitor therapy in vitro in combination with COX inhibitors in human, as well as in canine TCC cell lines [40]. However, MAP kinase inhibitors are not currently available for dogs.

Positive correlations between histological grade and COX-2 expression, as well as between COX-2 expression and the presence of BRAF mutation were presumed for canine TCC. Therefore, the aims of this study were to determine any correlation between histological grade and intensity of COX-2 expression, between the histological grade and the presence of BRAF mutation, as well as between the presence of BRAF mutation and the intensity of COX-2 expression in canine TCC. Furthermore, the differences in histological grade, intensity of COX-2 expression, and BRAF mutation between terriers and non-terrier breeds were evaluated.

## 2. Materials and Methods

### 2.1. Study Design

TCC biopsy samples from 65 dogs submitted for routine diagnostics were selected over a seven-year period (2012–2018) (Table 1 and Table 2). Inclusion criteria were: transmural biopsy (comprising mucosa, submucosa, and muscularis) and full information about breed, sex, and age of the dogs. The animals were between 6 and 14 years old (median 11 years). The cohort included 23 intact and 22 spayed females, as well as 12 intact and 8 castrated male dogs. Included were 15 terrier breeds according to the Fédération Cynologique Internationale (FCI) Group 3 (Section 1, large- and medium-sized Terriers: 1 Airedale terrier, 1 Fox terrier; Section 2, small-sized terriers: 5 Scottish terriers, 4 Jack Russel terriers, 3 West Highland white terriers; Section 4, toy tTerriers: 1 Yorkshire terrier) and 50 dogs of non-terrier breeds (21 mongrels, 4 beagles, 3 Shetland sheepdogs, 3 poodles, 3 Bernese mountain dogs, and 16 other breeds).

### 2.2. Histology

The formalin-fixed tissue specimens (min: 0.5 × 0.4 × 0.4 mm, max: 6.5 × 4.4 × 1.5 mm) were dehydrated through a graded series of ethanols (up to 96% ethanol) and embedded in paraplast (SAV-liquid Production GmbH, Flintsbach am Inn, Germany; PFNP-20-5858-1). Slices (3–4 μm) were mounted on coated slides (SuperFrost^®^ Plus, Menzel Gläser, Thermo Scientific, Waltham, MA USA). The standard hemalaun-eosin stain (HE) was performed [41]. Transitional cell carcinomas were diagnosed routinely and graded according to Meuten and Meuten [42] into low- or high-grade. Mitotic figures were counted in 10 high-power fields (HPFs; 400×; area: 68,700 µm^2^, Nikon Eclipse E200 microscope; Nikon, Tokyo, Japan) in areas with the highest mitotic activity, and the mean value was calculated.

Low-grade TCC was characterized by mild to moderate cellular atypia, mild nuclear abnormalities, rare to no mitoses, mild to no invasion of the submucosa with intact basement membrane, or no invasion into blood and lymphatic vessels.

In contrast, epithelial tumor cells of high-grade TCC showed loss of cell polarity, disorganized growth, marked cellular atypia, marked nuclear pleomorphism, or numerous mitoses. They penetrated the basement membrane and invaded deeper structures. Furthermore, they attached to and invaded blood or lymphatic vessels. In general, one characteristic feature of high-grade TCC is sufficient to define it as high-grade, but mostly numerous signs of malignancy coexist in canine TCC. The growth pattern was classified as papillary (projecting into the lumen) or non-papillary (sessile or flat) [42].

### 2.3. Immunohistochemistry

Tissue sections were mounted on SuperFrost slides. Pre-treatment at a high temperature (96 °C) with EDTA buffer (pH 9.0) was performed for 30 min. Cross-reacting monoclonal mouse anti-human COX-2 (1:100, clone cx-294, Dako, # 3617) diluted in antibody diluent (Zytomed, # ZUC025-100) served as the primary antibody. Canine TCC specimens were used as positive control tissues. Subsequently, sections were incubated overnight at 4 °C with the primary antibodies or a non-related isotype-matched antibody (negative control) [43]. As detection system, Dako EnVision+System-HRP (diaminobenzidine tetrahydrochloride (DAB)) (Dako, # K4006), was applied for 30 min at room temperature. All slides were finally developed in DAB (diaminobenzidine tetrahydrochloride, Dako, # K4006) for 10 min at room temperature and counterstained with hemalum.

Neoplastic and inflammatory cells were histologically identified. COX-2 expression was partly seen in the inflammatory and neoplastic cells, but not in the normal bladder epithelium. For this study, however, only neoplastic cells were further examined. In each specimen, the intensity of COX-2 expression of the neoplastic cells showed a great range, in some cases reaching from mild to marked in one sample. Thus, a modified version of the immunoreactive score (IRS) published by Hoffmann et al. [44] was used. For this, the percentages of positive neoplastic cells (PP) at the different staining intensities (SI) were assessed in the whole slide. The different staining intensities were scored with values ranging from 0–10 with the staining intensity being defined as: 0 = absence of staining, 0.5 = slight, 1 = mild, 5 = moderate, and 10 = most intense staining. The IRS was calculated as a sum of the PP multiplied with their respective SI scores, multiplied by the factor 1/100.

(1) $$IRS=\frac{1}{100}\overset{5}{\underset{n=1}{\sum }}\left\{PPnxSIn\right\}$$

*n* = index, PP = percentage of positive cells, SI = staining intensity.

The TCC were graded based on the COX-2 expression intensity (IRS) into minimal (IRS ≤ 0.9), mild (IRS > 0.9 to ≤ 3.3), moderate (IRS > 3.3 to ≤ 6.3), and marked (IRS > 6.3).

### 2.4. BRAF Mutation Analysis

DNA isolation from paraffin-embedded tissue was performed using a QIAamp DNA FFPE Tissue Kit (Qiagen, Hilden, Germany; # 56404). This kit uses a column-based method designed for purifying DNA from formalin-fixed, paraffin-embedded tissue sections. To do so, paraffin was dissolved in xylene. The sample was lysed under denaturing conditions with a short proteinase K digestion. After that, incubation at 90 °C reversed formalin cross-linking. DNA bound to the membrane of the column in a reaction tube, while contaminants flowed through. DNA was eluted and used for the subsequent PCR reaction. Exon 15 was examined for the presence of the mutation (BRAF variant c.1799T>A) by the TaqMan^®^ SNP assay (Applied Biosystems, Foster City, CA, USA, # 4332075) using FastStart Essential DNA Probes Master and LightCycler 480 II (Hoffmann-La Roche, Basel, Switzerland). The genotyping assay was performed using specific, provided PCR primers (forward primer TGGGACCCACTCCATCGA, reverse primer CATGAAGACCTCACAGTAAAAATAGGTGAT, no canine-specific primers) and probes (the probe sequence for detection of the wild-type allele was TAGCCACAGTGAAATC labelled with VIC™ fluorescent dye, the probe sequence for detection of the mutant allele was CCACAGAGAAATC with FAM™ fluorescent dye). Cycling conditions were 10 min at 95 °C, followed by 40 cycles of 15 s at 95 °C and 1 min at 60 °C.

### 2.5. Statistical Analyses

Statistical significance analyses were performed using GraphPad Prism Version 7.03 for Windows, GraphPad Software (La Jolla, CA, USA) www.graphpad.com. Data concerning correlations between breeds, histological grade, and BRAF mutation (nominal data) were tested using Fisher’s exact test. Ordinal data (IRS for COX-2 expression) were examined for the Gaussian distribution using the Shapiro–Wilk normality test. Non-parametric data (IRS for COX-2 expression) were verified using the Mann–Whitney U test. Values ≤0.05 were considered as significant.

## 3. Results

### 3.1. Histology

**Figure 1:**
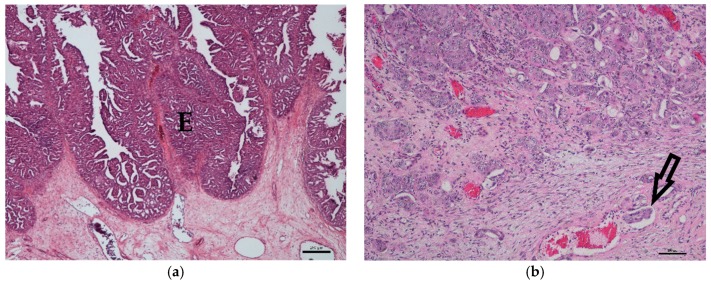
Histology of canine transitional cell carcinoma specimens. (**a**) Low-grade transitional cell carcinoma of an 11-year-old male mongrel. The epithelium (E) is markedly thickened and multi-layered, and the irregularly-arranged cells show mild anisocytosis and anisokaryosis. The basement membrane is intact (HE, bar = 250 μm). (**b**) High-grade transitional cell carcinoma of an eight-year-old castrated male mongrel with marked anisocytosis and anisokaryosis. The tumor intensively invades the submucosa, and additionally, an intravascular tumor cell embolus (arrow) is detectable (HE, bar = 100 μm).

Histological grading resulted in 20 (1/15 terriers, 19/50 non-terriers) low- and 45 (14/15 terriers, 31/50 non-terriers) high-grade TCC. Dogs with low-grade TCC (Figure 1a) were between seven and 14 years (median 11 years) old. The growth pattern was papillary in 13 and non-papillary in seven cases. A mild invasion of the submucosa without destruction of the basement membrane was seen in 11/20 and no invasion of the submucosa in 9/20 cases. Anisocytosis and anisokaryosis were mild, and few mitoses/HPF were counted. Vascular attachment was found in 6/20 cases, but vascular invasion was not seen in any of the samples.

Dogs with high-grade TCC (Figure 1b) were also, on average, 11 years old (range: 6–13 years). The growth pattern was non-papillary in 25 cases and papillary in 20 cases. The neoplasms massively invaded either the submucosa (6/45) or submucosa and muscularis (39/45). Anisocytosis and anisokaryosis were moderate to marked, and several mitoses/HPF were counted. Vascular attachment (35/45) and vascular invasion (23/45) were identified in many cases.

**Figure 2:**
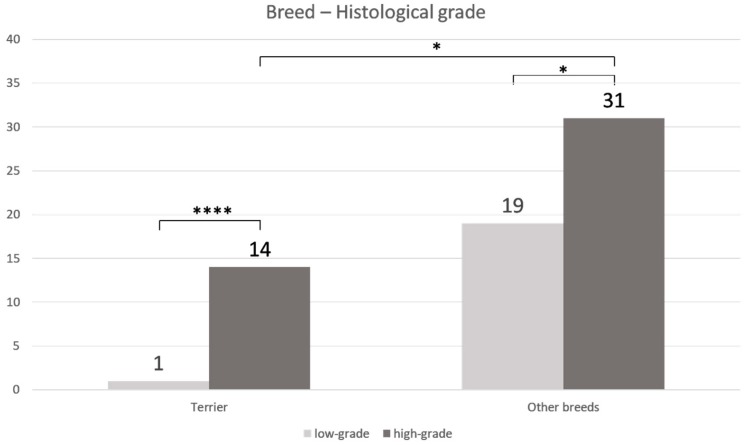
Correlation between histological grade and breed: A significantly higher percentage of high-grade transitional cell carcinoma (TCC) compared to low-grade TCC was observed in terriers (**** *p* < 0.0001) and in non-terriers (* *p* ≤ 0.05). Furthermore, a considerably higher percentage of high-grade transitional cell carcinoma was observed in terriers compared to non-terriers (* *p* ≤ 0.05) (Fisher’s exact test).

An age- or sex-related difference between low- and high-grade TCC was not detectable. In terriers (*p* < 0.0001), as well as in non-terriers (*p* ≤ 0.05), high-grade TCC was significantly more prevalent than low-grade TCC (Figure 2). Furthermore, high-grade TCC was considerably more often found in terriers than in non-terriers (*p* ≤ 0.05).

### 3.2. COX-2 Immunohistochemistry

**Figure 3:**
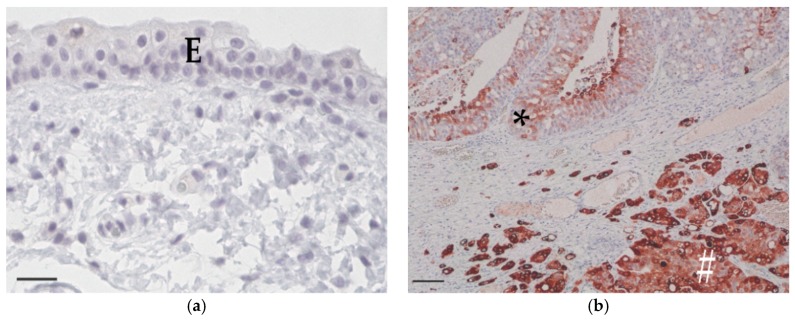

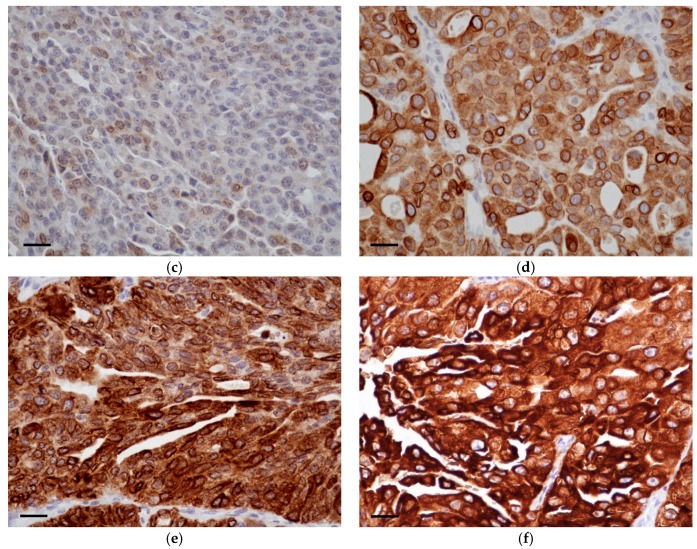
Cyclooxygenase-2 immunohistochemistry. (**a**) Normal bladder epithelium (E) of a 10-year-old female mongrel. There is no apparent cyclooxygenase-2 expression (COX-2, bar = 25 μm). (**b**) High-grade transitional cell carcinoma from an 11-year-old female Scottish terrier with an IRS of 7.6. Intensity of cyclooxygenase-2 expression varied from mild (*) to marked (#) within the specimen (COX-2, bar = 100 μm). (**c**) Area with minimal intensity of cyclooxygenase-2 expression in high-grade transitional cell carcinoma from an 11-year-old castrated female bracke (COX-2, bar = 25 μm). (**d**) Area with mild intensity of cyclooxygenase-2 expression in low-grade transitional cell carcinoma from a 12-year-old castrated female beagle (COX-2, bar = 25 μm). (**e**) Area with moderate intensity of cyclooxygenase-2 expression in high-grade transitional cell carcinoma from a 13-year-old castrated male mongrel (COX-2, bar = 25 μm). (**f**) Area with marked intensity of cyclooxygenase-2 expression in low-grade transitional cell carcinoma from an 11-year-old castrated male podenco (COX-2, bar = 25 μm).

Normal transitional cell epithelium was not labelled by anti-COX-2 antibody (Figure 3a). Inflammatory cells and neoplastic cells were positive. The intensity of COX-2 expression of the neoplastic cells varied strongly within each specimen (Figure 3b). Calculation of the COX-2 expression intensity (IRS) resulted in TCC being labelled as follows: nine minimal (IRS 0.2–0.9) (Figure 3c), 20 mild (IRS 1.0–3.3) (Figure 3d), 24 moderate (IRS 3.6–6.3) (Figure 3e), and 13 marked (IRS 6.6–9.8) (Figure 3f). COX-2 IRS was statistically not normally distributed. There was no age- or sex-related difference in COX-2 expression detectable. No significant (*p* = 0.9859) difference was found between COX-2 IRS of high-grade TCC (median 3.8, range: 0.2–9.8) and low-grade TCC (median 3.8, range: 0.3–7.8).

### 3.3. BRAF Mutation

**Figure 4:**
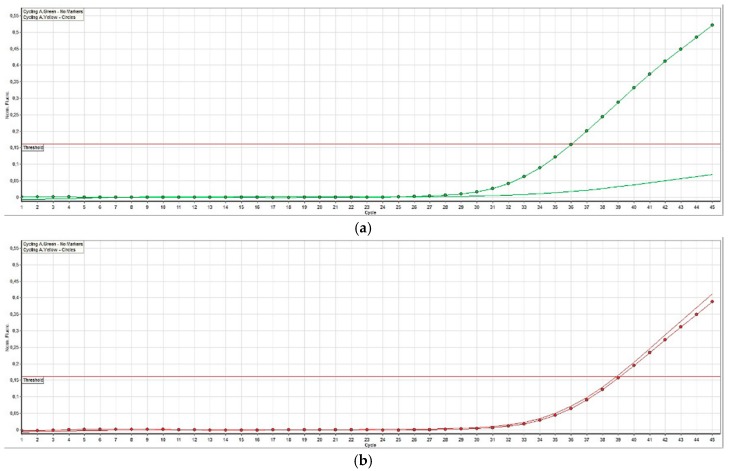
Fluorescent signals detected by the TaqMan^®^ SNP assay of a canine transitional cell carcinoma. (**a**) In transitional cell carcinoma without BRAF mutation, there is only a signal from the wild-type probe (homozygous thymine, dotted curve) and no signal from the variant probe (curve without dots). (**b**) In transitional cell carcinoma with BRAF mutation (heterozygous thymine and adenine), signal curves of both the wild-type probe (thymine, dotted curve) and the variant (adenine, curve without dots) can be seen.

**Figure 5:**
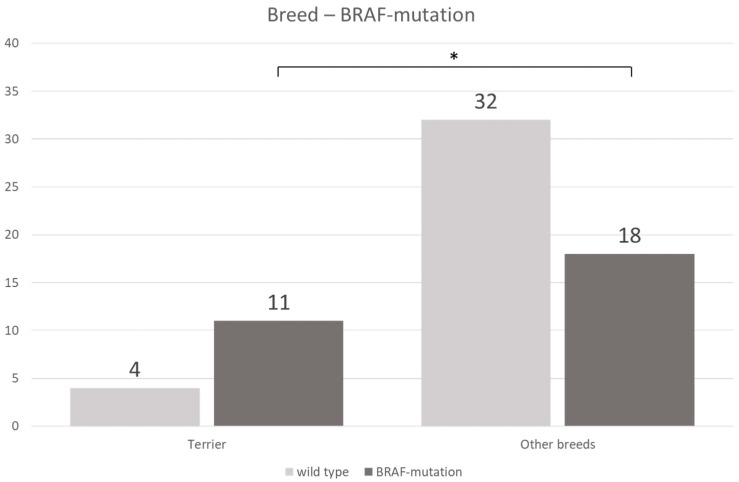
Correlation between breed and the presence or absence of BRAF mutation: BRAF mutation occurs significantly more often in transitional cell carcinoma in terriers than in transitional cell carcinoma in non-terriers (Fisher’s exact test, *p* ≤ 0.05).

**Figure 6:**
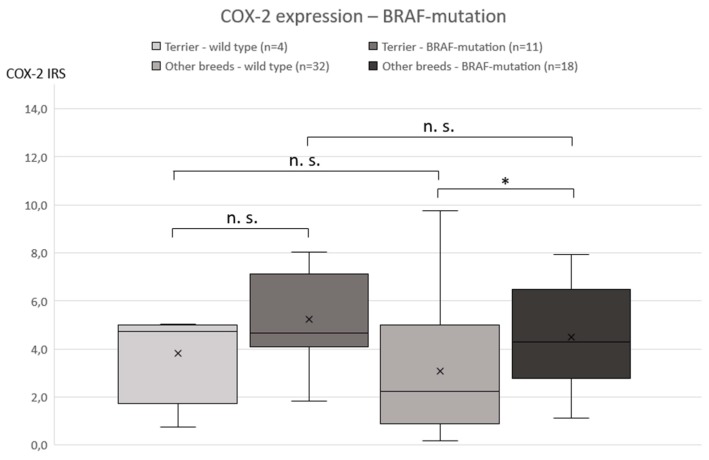
Correlation between the intensity of cyclooxygenase-2 expression and the presence or absence of BRAF mutation: a significant difference in the cyclooxygenase-2 expression was only detected in non-terrier breeds comparing BRAF mutation-positive and -negative transitional cell carcinoma (Mann–Whitney *U*-test, *p* ≤ 0.05).

Genetic testing of 65 TCC showed BRAF mutation in 29 cases (11/15 terriers, 18/50 non-terriers) and the wild-type in 36 dogs (4/15 terriers, 32/50 non-terriers) (Figure 4). There was no significant (*p* = 0.2912) difference in the prevalence of BRAF mutation between low- and high-grade TCC. BRAF mutation was significantly (*p* ≤ 0.05) more often found in TCC of Terriers than in TCC of non-terrier breeds (Figure 5). In terriers, COX-2 expression intensity did not show a significant difference (*p* = 0.3915) between neoplasms with or without BRAF mutation. In contrast, in non-terrier breeds, TCC with BRAF mutation had a significantly (*p* ≤ 0.05) higher COX-2 expression than TCC without BRAF mutation. TCC without BRAF mutation had a higher COX-2 expression in terriers than TCC without BRAF mutation had in non-terriers, but this difference was not significant (*p* = 0.4154). There was also no significant (*p* = 0.0579) difference in the intensity of COX-2 expression between BRAF mutation-positive TCC in terriers and BRAF mutation-positive TCC in non-terriers (Figure 6).

**Figure 7:**
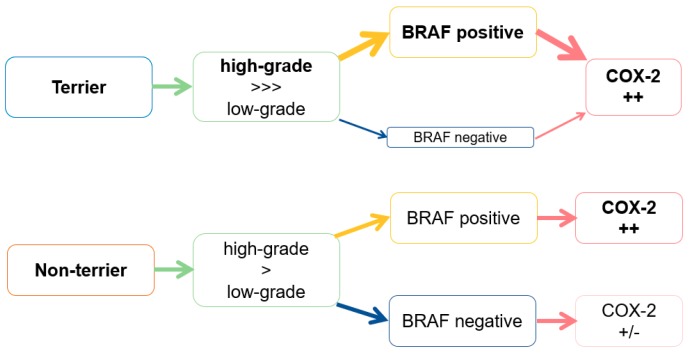
Interpretation algorithm for BRAF mutation in terriers and non-terrier breeds in correlation with the histological grade and cyclooxygenase-2 expression.

In conclusion, this interpretation algorithm (Figure 7) can be derived from the data of transmural biopsies in our study: In cases of TCC with BRAF mutation in terriers, a high-grade tumor with increased COX-2 expression is most likely. Cases of TCC without BRAF mutation are rare in the analyzed terrier breeds. Irrespective of the BRAF mutation status, in the case of TCC in a non-terrier, high-grade TCC, is more likely than low-grade TCC, just as it is in terriers. However, since terriers suffer significantly more often from high-grade TCC than non-terriers, it cannot be assumed that the high-grade morphology in non-terrier TCC is the most likely. Therefore, the histological grade of TCC in non-terriers should always be verified in addition by histological examination. As in terriers, an increased COX-2 expression in TCC in non-terriers is only likely if a positive BRAF mutation is detected.

## 4. Discussion

To the authors’ knowledge, this is the first study in dogs investigating the histological grade of TCC, the intensity of COX-2 expression, and the presence of the BRAF mutation with a special focus on breed predilection of terriers.

Although canine and human TCC are largely and histopathologically very similar [45], there are some remarkable differences regarding the sex predisposition, histological grade, and prevalence of BRAF mutation, as well as a notable correlation of histological grade and intensity of COX-2 expression. A known sex predisposition for female dogs suffering from TCC [3] was confirmed by the present study. In humans, a gender predisposition is described for men [16]. Furthermore, canine TCC shows mostly high-grade malignancy [2,42]. This was confirmed by the present data and is contrary to the situation in humans where the majority of TCC is classified as superficial low-grade [3]. Another difference between both species is the prevalence of BRAF mutation in TCC. While infrequently in human TCC (<1%) [31], BRAF mutation is often detected in canine TCC [26,27,28,29,39]. Additional BRAF mutations in exons 11 and 14 were reported in human cancers [46], but until now, not in dogs. In humans, a significantly higher intensity of COX-2 expression is known in high-grade TCC [17,19]. In the present study, we found no considerable difference in the intensity of COX-2 expression between low- and high-grade canine TCC. However, a significant positive correlation was found between the presence of BRAF mutation and the intensity of COX-2 expression in TCC in non-terrier breeds. This finding is consistent with the results of a study in humans describing higher COX-2 expression in BRAF mutation-positive colorectal cancer than in BRAF mutation-negative intestinal tissue [38]. The authors of that study suggested that the correlation between BRAF mutation and COX-2 is mediated by insulin-like growth factor receptor 1 (IGF-1R) as shown in human pancreatic carcinomas, where IGF-1R is enhanced by the BRAF mutation and mediates COX-2 expression selectively via the MAPK/(Erk-1/2) pathway [47]. Whether this mechanism is also present in canine TCC requires verification. It is possible that the correlation of BRAF mutation and the intensity of COX-2 expression in terrier breeds would have reached significance if more cases had been included in this group. Because of the high heterogeneity of the studies addressing COX-2 expression intensity, which has already been discussed, it seems difficult to compare the various results. It is therefore still questionable if COX-2 expression is a predictive factor for the response of TCC to COX-2 inhibitors [48]. Additional research, including the development of in vitro cell systems, is needed to determine if COX-2 expression can be used as a reliable prognostic factor and as a definite therapeutic target in animal cancers [48]. Overall, the use of COX-2 inhibitors in the treatment of canine, as well as of human TCC is common, and the efficiency has been proven in numerous studies [40,49,50,51].

Terriers have a well-documented breed predisposition for TCC [1]. It has to be taken into consideration that histological grading of urinary bladder biopsies requires transmural samples of sufficient size to evaluate intramural and intravascular invasion [42]. In endoscopy, sampling of biopsies with sufficient size is sometimes not possible. The present study shows that high-grade TCC is significantly more common in terriers than in non-terrier breeds. It can be concluded from these findings that high-grade TCC can be assumed as statistically most likely in terriers, even if histological grading may not be possible if the size of the specimen is too small.

Our results confirm the results by Pantke et al. who reported a significantly higher prevalence of BRAF mutation in TCC in terriers compared to TCC in non-terrier breeds [6]. This may be the reason for the high breed-associated risk regarding TCC in terriers [1] and may result from a specific genetic polymorphism in this breed. Such polymorphisms are responsible for the gender predisposition regarding BRAF mutation of melanomas in humans [52]. Whether such polymorphisms exist in dogs and if they make the BRAF gene susceptible to mutagenic factors could be the subject of further studies. However, Aupperle-Lellbach et al. (18 terriers and 48 non-terrier breeds) and Decker et al. (18 terriers, 38 non-terrier breeds, and 8 non-specified breeds) did not identify a breed disposition for BRAF mutation [26,27]. In another study by Maeda et al., only one terrier suffering from TCC was included [53], and two further studies by Mochizuki et al. did not mention the breeds that were included [28,29].

The lower percentage of TCC with BRAF mutation (48%) found in the present study compared to previous studies [26,27,28,29,53] may result from the lower number of included terriers in the study cohort. Moreover, it must be considered that the genetic pool for certain breeds in the U.S. does not necessarily parallel the genetic pool in Europe, and so, comparison of studies from the U.S. and Europe may be difficult.

Due to the high specificity (100%) of BRAF mutation in canine TCC [26], analysis is recommended in all breeds in the case of questionable, non-diagnostic cytological or histological samples. This avoids further (necessary) invasive sampling. Due to the high sensitivity of BRAF mutation for terriers in our study (73%) and the breed predisposition of terriers to suffer from TCC, testing for the presence of BRAF mutation in this breed is recommended as an additional screening test independent of age [54]. Urine can be used for this purpose. It must be considered that only a positive result is diagnostic for the presence of TCC. If BRAF mutation cannot be detected, the tumor is either not caused by this mutation, no mutated cells are present in the specimen, or there is no TCC. Nevertheless, testing for the presence of BRAF mutation can be used as a first, non-invasive test for the diagnosis of canine TCC.

## 5. Conclusions

Despite some similarities, there are also marked differences between canine and human TCC. Testing for BRAF mutation in canine TCC is a sensitive diagnostic method especially in terriers (73%) and recommended as a screening test. However, contrary to humans, the detection of BRAF mutation in canine TCC is not a predictor for the histological grade, and a positive correlation between histological grade and intensity of COX-2 expression was not found. The clinical and prognostic relevance of the increased intensity of COX-2 expression in TCC with BRAF mutation in non-terrier breeds has to be clarified in further studies.

## Figures and Tables

**Table 1:** Terriers: signalment, histological grade, cyclooxygenase-2 expression, and BRAF mutation in transitional cell carcinoma (*n* = 15).

Breed	Mean Age (Years)	Sex	Histological Grade	Median COX-2 IRS	BRAF Mutation
Scottish terrier (*n* = 5)	10 ± 2	3 F, 1 FN, 1 M	5 high	4.5 (range: 0.8–7.6)	4+ 1−
Jack Russel terrier (*n* = 4)	12 ± 1	3 F, 1 FN	4 high	4.8 (range: 4.5–5.1)	3+ 1−
West Highland white terrier (*n* = 2)	11 ± 2	1 FN, 1 M	1 high 1 low	5.8 (range: 4.8–6.7)	1+ 1−
Airedale terrier (*n* = 1)	10	FN	high	4.1	+
Fox terrier (*n* = 1)	12	FN	high	3.8	+
Welsh terrier (*n* = 1)	12	FN	high	4.7	−
Yorkshire terrier (*n* = 1)	11	F	high	7.1	+

+ = BRAF mutation positive, − = BRAF mutation negative, COX = cyclooxygenase, F = female, FN = neutered female, IRS = immunoreactive score, M = male, MN = neutered male.

**Table 2:** Non-terrier breeds: signalment, histological grade, cyclooxygenase-2 expression, and BRAF mutation in transitional cell carcinoma (*n* = 50).

Breed	Mean Age (Years)	Sex	Histological Grade	Median COX-2 IRS	BRAF Mutation
Mongrel (*n* = 21)	11 ± 2	7 F, 6 FN, 4 M, 4MN	13 high 8 low	4.0 (range: 1.4–8.1)	9+ 12−
Beagle (*n* = 4)	10 ± 2	1 F, 3 FN	3 high 1 low	1.9 (range: 1.3–7.9)	3+ 1−
Bernese mountain dog (*n* = 3)	9 ± 2	1 FN, 2 M	2 high 1 low	5.2 (range: 0.5–6.9)	1+ 2−
Cocker spaniel (*n* = 3)	10 ± 3	1 F, 1 FN, 1 MN	2 high 1 low	0.7 (range: 0.4–1.2)	3−
Poodle (*n* = 3)	11 ± 1	1 F, 1 FN, 1 M	2 high 1 low	3.3 (range: 3.0–7.8)	2+ 1−
Shetland sheepdog (*n* = 3)	10 ± 2	3 F	1 high 2 low	1.9 (range: 0.3–2.3)	1+ 2−
Australianshepherd (*n* = 2)	10 ± 1	1 F, 1 FN	1 high 1 low	4.4 (range: 3.8–5.0)	2−
Small Münsterländer (*n* = 1)	11	F	low	5.0	−
Rottweiler (*n* = 1)	10	M	low	1.0	−
Podenco (*n* = 1)	11	MN	low	7.8	+
Siberian husky (*n* = 1)	12	MN	low	1.1	+
German wirehaired pointer (*n* = 1)	8	F	high	0.8	−
Great dane (*n* = 1)	7	M	high	0.9	−
Bracke (*n* = 1)	11	FN	high	0.2	−
French bulldog (*n* = 1)	10	F	high	4.1	−
Basset (*n* = 1)	12	FN	high	9.8	−
Bichon frise (*n* = 1)	11	MN	high	0.3	−
Border collie (*n* = 1)	12	M	high	2.0	−

+ = BRAF mutation positive, − = BRAF mutation negative, COX = cyclooxygenase, F = female, FN = neutered female, IRS = immunoreactive score, M = male, MN = neutered male.

## References

[1] Fulkerson C.M., Knapp D.W. (2015). Management of transitional cell carcinoma of the urinary bladder in dogs: A review. Vet. J..

[2] Mutsaers A.J., Widmer W.R., Deborah W., Knapp D.W. (2003). Canine Transitional Cell Carcinoma. J. Vet. Intern Med..

[3] Knapp D.W., Glickman N.W., DeNicola D.B., Bonney P.L., Lin T.L., Glickman L.T. (2000). Naturally-occurring canine transitional cell carcinoma of the urinary bladder. A relevant model of human invasive bladder cancer. Urol. Oncol..

[4] Knapp D.W., Ramos-Vara J.A., Moore G.E., Dhawan D., Bonney P.L., Young K.E. (2014). Urinary bladder cancer in dogs, a naturally occurring model for cancer biology and drug development. Ilar J..

[5] Norris A.M., Laing E.J., Valli V.E., Withrow S.J., Macy D.W., Ogilvie G.K., Tomlinson J., McCaw D., Pidgeon G., Jacobs R.M. (1992). Canine bladder and urethral tumors: A retrospective study of 115 cases (1980–1985). J. Vet. Intern. Med..

[6] Pantke P. (2018). Diagnosis and treatment of transitional cell carcinoma of the lower urinary tract in the dog. Kleintierprax.

[7] Knapp D.W., Ruple-Czerniak A., Ramos-Vara J.A., Naughton J.F., Fulkerson C.M., Honkisz S.I. (2016). A nonselective cyclooxygenase inhibitor enhances the activity of vinblastine in a naturally-occurring canine model of invasive urothelial carcinoma. Bladder Cancer.

[8] Knottenbelt C., Mellor D., Nixon C., Thompson H., Argyle D.J. (2006). Cohort study of COX-1 and COX-2 expression in canine rectal and bladder tumours. J. Small Anim. Pract..

[9] Mohammed S.I., Bennett P.F., Craig B.A., Glickman N.W., Mutsaers A.J., Snyder P.W., Widmer W.R., DeGortari A.E., Bonney P.L., Knapp D.W. (2002). Effects of the cyclooxygenase inhibitor, piroxicam, on tumor response, apoptosis, and angiogenesis in a canine model of human invasive urinary bladder cancer. Cancer Res..

[10] Carvalho S., Stoll A.L., Priestnall S.L., Suarez-Bonnet A., Rassnick K., Lynch S., Schoepper I., Romanelli G., Buracco P., Atherton M. (2017). Retrospective evaluation of COX-2 expression, histological and clinical factors as prognostic indicators in dogs with renal cell carcinomas undergoing nephrectomy. Vet. Comp. Oncol..

[11] Mohammed S.I., Khan K.N., Sellers R.S., Hayek M.G., DeNicola D.B., Wu L., Bonney P.L., Knapp D.W. (2004). Expression of cyclooxygenase-1 and 2 in naturally-occurring canine cancer. Prostaglandins Leukot. Essent. Fat. Acids.

[12] Khan K.N., Knapp D.W., Denicola D.B., Harris R.K. (2000). Expression of cyclooxygenase-2 in transitional cell carcinoma of the urinary bladder in dogs. Am. J. Vet. Res..

[13] Lee J.Y., Tanabe S., Shimohira H., Kobayashi Y., Oomachi T., Azuma S., Ogihara K., Inokuma H. (2007). Expression of cyclooxygenase-2, P-glycoprotein and multi-drug resistance-associated protein in canine transitional cell carcinoma. Res. Vet. Sci..

[14] Mutsaers A.J., Mohammed S.I., DeNicola D.B., Snyder P.W., Glickman N.W., Bennett P.F., de Gortari A.E., Bonney P.L., Knapp D.W. (2005). Pretreatment tumor prostaglandin E2 concentration and cyclooxygenase-2 expression are not associated with the response of canine naturally occurring invasive urinary bladder cancer to cyclooxygenase inhibitor therapy. Prostaglandins Leukot. Essent. Fat. Acids.

[15] Hurst E.A., Pang L.Y., Argyle D.J. (2019). The selective cyclooxygenase-2 inhibitor mavacoxib (Trocoxcil™) exerts anti-tumour effects in-vitro independent of cyclooxygenase-2 expression levels. Vet. Comp. Oncol..

[16] Herr H.W., Shipley W.U., Bajorin D.F., Hellman S., Rosenberg S.A. (2001). Cancer of the bladder. DeVita VT.

[17] Shariat S.F., Matsumoto K., Kim J., Ayala G.E., Zhou J.H., Jian W., Benedict W.F., Lerner S. (2003). Correlation of cyclooxygenase-2 expression with molecular markers, pathological features and clinical outcome of transitional cell carcinoma of the bladder. J. Urol..

[18] Agrawal U., Kumari N., Vasudeva P., Mohanty N.K., Saxena S. (2018). Overexpression of COX2 indicates poor survival in urothelial bladder cancer. Ann. Diagn. Pathol..

[19] Kömhoff M., Guan Y., Shappell H.W., Davis L., Jack G., Shyr Y., Koch M.O., Shappell S.B., Breyer M.D. (2000). Enhanced expression of cyclooxygenase-2 in high grade human transitional cell bladder carcinomas. Am. J. Pathol..

[20] Tabriz H.M., Olfati G., Ahmadi S.A., Yusefnia S. (2013). Cyclooxygenase-2 expression in urinary bladder transitional cell carcinoma and its association with clinicopathological characteristics. Asian Pac. J. Cancer Prev..

[21] Manoharan M., Soloway M.S. (2005). Optimal management of the T1G3 bladder cancer. Urol. Clin. N. Am..

[22] Reisz P.A., Laviana A.A., Chang S.S. (2018). Management of High-grade T1 Urothelial Carcinoma. Curr. Urol. Rep..

[23] Chou R., Buckley D., Fu R., Gore J.L., Gustafson K., Griffin J., Grusing S., Selph S. (2015). AHRQ comparative effectiveness reviews. Emerging Approaches Diagnose and Treatment Non-Muscle-Invasive Bladder Cancer [Internet] 2015.

[24] Burkhari N., Al-Shamsi H.O., Azam F. (2018). Update on the Treatment of Metastatic Urothelial Carcinoma. Sci. World J..

[25] Downward J. (2003). Targeting RAS signalling pathways in cancer therapy. Nat. Rev. Cancer.

[26] Aupperle-Lellbach H., Grassinger J., Hohloch C., Kehl A., Pantke P. (2018). Diagnostic value of the BRAF variant V595E in urine samples, smears and biopsies from canine transitional cell carcinoma. Tierarztl. Prax. K..

[27] Decker B., Parker H.G., Dhawan D., Kwon E.M., Karlins E., Davis B.W., Ramos-Vara J.A., Bonney P.L., McNiel E.A., Knapp D.W. (2015). Homologous mutation to human BRAF V600E is common in naturally occurring canine bladder cancer—Evidence for a relevant model system and urine-based diagnostic test. Mol. Cancer Res..

[28] Mochizuki H., Kennedy K., Shapiro S.G., Breen M. (2015). BRAF Mutations in canine cancers. PLoS ONE.

[29] Mochizuki H., Shapiro S.G., Breen M. (2015). Detection of BRAF Mutation in urine DNA as a molecular diagnostic for canine urothelial and prostatic carcinoma. PLoS ONE.

[30] Dhillon A., Hagan S., Rath O., Kolch W. (2007). Map kinase signalling pathways in cancer. Oncogene.

[31] Mochizuki H., Breen M. (2015). Comparative aspects of BRAF mutations in canine cancers. Vet. Sci..

[32] Boulalas I., Zaravinos A., Delakas D., Spandidos D.A. (2009). Mutational analysis of the BRAF gene in transitional cell carcinoma of the bladder. Int. J. Biol. Markers.

[33] Marranci A., Jiang Z., Vitiello M., Guzzolino E., Comelli L., Sarti S., Lubrano S., Franchin C., Echevarría-Vargas I., Tuccoli A. (2017). The landscape of BRAF transcript and protein variants in human cancer. Mol. Cancer.

[34] Cancer Genome Atlas Network (2015). Genomic classification of cutaneous melanoma. Cell.

[35] Kimura E.T., Nikiforova M.N., Zhu Z., Knauf J.A., Nikiforov Y.E., Fagin J.A. (2003). High prevalence of BRAF mutations in thyroid cancer: Genetic evidence for constitutive activation of the RET/PTC-RAS-BRAF signaling pathway in papillary thyroid carcinoma. Cancer Res..

[36] Rajagopalan H., Bardelli A., Lengauer C., Kinzler K.W., Vogelstein B., Velculescu V.E. (2002). Tumorigenesis: RAF/RAS oncogenes and mismatch-repair status. Nature.

[37] Cancer Genome Atlas Research Network (2014). Comprehensive molecular profiling of lung adenocarcinoma. Nature.

[38] Jin M., Long Z.W., Yang J., Lin X. (2018). Correlations of IGF-1R and COX-2 Expressions with Ras and BRAF genetic mutations, Clinicopathological Features and Prognosis of Colorectal Cancer Patients. Pathol. Oncol. Res..

[39] Mochizuki H., Breen M. (2017). Sequence analysis of RAS and RAF mutation hot spots in canine carcinoma. Vet. Comp. Oncol..

[40] Bourn J., Cekanova M. (2018). Cyclooxygenase inhibitors potentiate receptor tyrosine kinase therapies in bladder cancer cells in vitro. Drug Des. Devel. Ther..

[41] Fischer A.H., Jacobson K.A., Rose J., Zeller R. (2008). Hematoxylin and eosin staining of tissue and cell sections. Csh Protoc..

[42] Meuten D.J., Meuten T.L.K., Meuten D.J. (2017). Tumors of the Urinary System. Tumors of Domestic Animals.

[43] Ramos-Vara J.A., Miller M.A. (2013). When tissue antigens and antibodies get along: Revisiting the technical aspects of immunohistochemistry—The red, brown, and blue technique. Vet. Pathol..

[44] Hoffmann C., Bazer F.W., Klug J., Aupperle H., Ellenberger C., Schoon H.A. (2009). Immunohistochemical and histochemical identification of proteins and carbohydrates in the equine endometrium Expression patterns for mares suffering from endometrosis. Theriogenology.

[45] De Brot S., Robinson B.D., Scase T., Grau-Roma L., Wilkinson E., Boorjian S.A., Gardner D., Mongan N.P. (2018). The dog as an animal model for bladder and urethral urothelial carcinoma: Comparative epidemiology and histology. Oncol. Lett..

[46] Davies H., Bignell G.R., Cox C., Stephens P., Edkins S., Clegg S., Teague J., Woffendin H., Garnett M.J., Bottomley W. (2002). Mutations of the BRAF gene in human cancer. Nature.

[47] Stoeltzing O., Liu W., Fan F., Wagner C., Stengel K., Somcio R.J., Reinmuth N., Parikh A.A., Hicklin D.J., Ellis L.M. (2007). Regulation of cyclooxygenase-2 (COX-2) expression in human pancreatic carcinoma cells by the insulin-like growth factor-I receptor (IGF-IR) system. Cancer Lett..

[48] Doré M. (2011). Cyclooxygenase-2 expression in animal cancers. Vet. Pathol..

[49] Allstadt S.D., Rodriguez C.O., Boostrom B., Rebhun R.B., Skorupski K.A. (2015). Randomized phase III trial of piroxicam in combination with mitoxantrone or carboplatin for first-line treatment of urogenital tract transitional cell carcinoma in dogs. J. Vet. Intern. Med..

[50] Knapp D.W., Richardson R.C., Chan T.C., Bottoms G.D., Widmer W.R., DeNicola D.B., Teclaw R., Bonney P.L., Kuczek T. (1994). Piroxicam therapy in 34 dogs with transitional cell carcinoma of the urinary bladder. J. Vet. Intern. Med..

[51] Arantes-Rodrigues R., Pinto-Leite R., Ferreira R., Neuparth M.J., Pires M.J., Gaivão I., Palmeira C., Santos L., Colaço A., Oliveira P. (2013). Meloxicam in the treatment of in vitro and in vivo models of urinary bladder cancer. Biomed. Pharmacother..

[52] Meyer P., Sergi C., Garbe C. (2003). Polymorphisms of the BRAF gene predispose males to malignant melanoma. J. Carcinog..

[53] Maeda S., Tomiyasu H., Tsuboi M., Inoue A., Ishihara G., Uchikai T., Chambers J.K., Uchida K., Yonezawa T., Matsuki N. (2018). Comprehensive gene expression analysis of canine invasive urothelial bladder carcinoma by RNA-Seq. BMC Cancer.

[54] Breen M., Wiley C. (2018). Flüssigbiopsie—Die Zukunft der Tumordiagnostik. Vet. Focus.

